# Perioperative Changes in Hemostatic Properties as Assessed by Multiplate, Siemens PFA-200, and ROTEM—A Comparative Study

**DOI:** 10.3390/jcm14051640

**Published:** 2025-02-28

**Authors:** Zrinka Starcevic, Martina Zrno-Mihaljevic, Hrvoje Gasparovic, Marijan Pasalic, Mirna Petricevic, Klaus Goerlinger, Mate Petricevic

**Affiliations:** 1 Department of Cardiac Surgery, University Hospital Centre Zagreb, 10000 Zagreb, Croatia; zrinkastarcevic@gmail.com (Z.S.); martina_zrno@yahoo.com (M.Z.-M.); hgasparovic@gmail.com (H.G.); 2 Department of Cardiovascular Diseases, University Hospital Centre Zagreb, 10000 Zagreb, Croatia; marijan.pasalic@yahoo.com; 3 Department of Health Studies, University of Split, 21000 Split, Croatia; petricevic.mirna@gmail.com; 4 Department of Anesthesiology and Intensive Care Medicine, University Hospital Essen, University Duisburg-Essen, 45127 Essen, Germany; kgoerlinger@werfen.com; 5 Medical Department, Tem Innovations, 81829 Munich, Germany; 6 School of Medicine, University Hospital of Split, University of Split, 21000 Split, Croatia

**Keywords:** coronary artery bypass graft surgery, bleeding, transfusion, platelet function testing, rotational thromboelastometry, impedance aggregometry

## Abstract

**Objectives:** This study sought to determine the platelet function and viscoelastic blood properties in the pre- and postoperative period using three different point-of-care (POC) devices (Multiplate^®^, Siemens PFA-200^®^ and ROTEM^®^). We aimed to investigate the association between preoperative POC test results and bleeding outcomes. Postoperative changes in blood hemostatic properties were also evaluated, as well as the agreement between two platelet function analyzers and rotational thromboelastometry parameters. **Methods:** The study was conducted in a prospective observational fashion. Patients undergoing elective coronary artery bypass graft surgery (CABG) were enrolled. Hemostatic blood properties were assessed using three different POC devices; two platelet function analyzers were used: (1) Impedance aggregometry (Multiplate^®^) with the arachidonic acid (ASPI) test and adenosine diphosphate (ADP) test. (2) The Siemens INNOVANCE^®^ PFA-200 System with the following assays: the PFA Collagen/EPI test, PFA Collagen/ADP test, and the INNOVANCE^®^ PFA P2Y test. Viscoelastic blood properties were assessed using ROTEM^®^ delta (TEM Innovations GmbH, Munich, Germany). POC tests were performed simultaneously at two different time points: (1) before surgery and (2) on postoperative day 4, respectively. The primary outcome was defined as amounts of perioperative bleeding and transfusion requirements, classified according to the universal definition for perioperative bleeding (UDPB) score. **Results:** The study recruited a total number of 63 patients undergoing elective isolated coronary artery bypass graft surgery (CABG). Based on the packed red blood cell (PRBC) transfusion requirements, patients with excessive bleeding were not just only frequently transfused (87.5% vs. 48.9%, *p* = 0.007) but were also transfused with higher amounts of PRBCs (1338.75 mL ± SD 1416.49 vs. 289.36 mL ± 373.07, *p* < 0.001). The FIBTEM A30 results significantly correlated with excessive bleeding (Correlation Coefficient Rho = −0.280, *p* = 0.028). Regression analysis revealed FIBTEM A 30 as a strongest predictor of 24 h chest tube output (CTO) (R Square 0.108, *p* = 0.009). The receiver operating characteristics curve (ROC) analysis showed that a preoperative FIBTEM A30 < 10.86 mm predicted excessive bleeding with 94% sensitivity and 50% specificity (ROC AUC 68.4%). The multiplate ASPI test results were significantly higher (35.24 AUC ± SD 22.24 vs. 19.43 AUC ± SD 10.74) and the proportion of Aspirin responders was significantly lower (42.4% vs. 76.7%, *p* = 0.006) in patients considered to have insignificant bleeding. On postoperative day 4, we found platelet hyperreactivity in the ASPItest coupled with a ROTEM-documented shift towards hypercoagulability. **Conclusions:** Modern hemostatic management and perioperative antiplatelet therapy (APT) administration/discontinuation management should be guided by thromboelastometry and platelet function testing. Prospective interventional trials are necessary to validate such an approach in multicentric studies.

## 1. Introduction

### 1.1. Hemostatic Point-of-Care Testing (HPOCT) and Bleeding Outcomes

Perioperative excessive bleeding after cardiac surgery occurs with an approximate incidence of about 20% [1] and with a re-exploration rate for excessive bleeding of up to 9% [2].

Hemostatic alterations certainly play a major role in cardiac surgery, particularly when it comes to bleeding complications and transfusion requirements.

Preoperatively, platelet dysfunction with a pronounced and prolonged platelet inhibitory response to preoperatively administered antiplatelet therapy (APT) certainly influences both bleeding complications and transfusion requirements. Hemostatic alterations in secondary hemostasis (coagulation) may also play an important role in terms of the occurrence of bleeding complications and transfusion requirements. Hemostatic alterations are preoperatively influenced by individual platelet function, viscoelastic blood properties, and both antiplatelet and anticoagulant medications. In cardiac surgery patients, these hemostatic alterations are further corroborated by the hemostatic alterations induced by cardiopulmonary bypass (CPB) and hypothermia which are usually required for cardiac surgery procedures.

Although there are many devices and different tests analyzing hemostatic blood properties available, the transfusion of blood and blood products is still mostly empiric and varies among institutions and even among specialists. The administration of allogenic blood products presents various risks to the patient [3,4,5], increases expenses [6,7] and the morbidity and mortality of cardiac surgery patients, and are directly proportional to the number of units of PRBCs transfused [8,9].

The standard laboratory assessments frequently utilized in the management of perioperative bleeding include prothrombin time (or international normalized ratio), activated partial thromboplastin time (aPTT), fibrinogen levels (using the Clauss method and its variations), and platelet count. With the exception of the platelet count, these tests necessitate the separation of plasma from whole blood prior to analysis, resulting in an average turnaround time of approximately 30 to 90 min [10]. The findings from these tests prompt requests for particular blood components. The selection of hemostatic treatments also influences the delay before intervention. Thawing Fresh Frozen Plasma (FFP) and cryoprecipitate usually takes about 30 to 60 min, whereas platelet concentrates and thawed plasma require less time. Additionally, factor concentrates, like fibrinogen concentrate, can be delivered quickly, within approximately 10 min [10]. When combined, the lengthy turnaround times and the preparation required for transfusion products render timely intervention unfeasible in the rapidly changing conditions of cardiac surgery patients. In contrast, point-of-care (POC) testing offers a significant benefit over conventional laboratory tests because of its quicker turnaround times, allowing for prompt decisions regarding coagulation interventions [11]. This is especially crucial for intraoperative measurements, which inform clinicians’ decisions regarding transfusion administration. In the early postoperative period, particularly in cases of significant bleeding, it is vital for clinicians to identify the cause of the bleeding and clearly differentiate between surgical bleeding that requires re-exploration and bleeding resulting from a hemostatic disorder that demands immediate non-surgical interventions. The turnaround time for traditional hemostatic laboratory tests is often too lengthy for the fast-paced environment of cardiac surgeries. Since this turnaround time exceeds the necessary “time to react,” we can conclude that conventional laboratory testing has limited utility in these situations. Quick decision-making is crucial in cardiac surgery, and bedside point-of-care devices can be invaluable due to their rapid turnaround time of just a few minutes. One of the significant challenges in effectively managing hemostasis, especially in cardiac surgery, is the delay in obtaining coagulation test results from central laboratories [12]. Additionally, point-of-care testing was shown to be superior to traditional laboratory tests in predicting postoperative bleeding [13]. In light of the points mentioned earlier, it appears that point-of-care testing for hemostasis plays a crucial role in hemostatic management and has the potential to enhance patient outcomes by transitioning from a “one-size-fits-all” strategy to a more personalized approach.

### 1.2. HPOCT and Perioperative Changes in Platelet Reactivity and Viscoelastic Blood Properties

Residual platelet reactivity (RPR) following coronary artery bypass graft surgery (CABG) might be related to thrombotic complications and major ischemic cardiac events.

The variability in platelet response during antiplatelet therapy (APT) can range from significant platelet inhibition to elevated levels of reactivity, which are frequently viewed as resistance to APT. This resistance is a crucial factor that can impact clinical outcomes and should significantly guide decisions regarding the initiation or cessation of APT. Although it is generally simpler to choose to move forward with surgery without halting medication when high reactivity is present, there is considerably less understanding of how to manage high reactivity in the postoperative period [14]. The importance of and difficulties associated with managing APT resistance in patients undergoing cardiac surgery have already been addressed by our working group [14].

There are multiple point-of-care platelet function analyzers that utilize various measurement methods and can deliver drug-specific platelet function assessments. The availability of various point-of-care (POC) platelet function analyzers, along with the absence of standardization in trial design and outcome definitions, complicates the aggregation of evidence. Furthermore, it remains unclear whether postoperative platelet function hyperactivity is coupled with hypercoagulability in the early postoperative period and whether this should influence the decision on a more aggressive antiplatelet therapy regime following CABG.

This study sought to determine platelet function and viscoelastic blood properties in the pre- and postoperative period, using three different POC devices. Postoperative changes in blood hemostatic properties were evaluated along with the agreement between two platelet function analyzers and rotational thromboelastometry parameters.

The aims of this study were twofold:

(1) To evaluate whether preoperative POC testing with two platelet function analyzers and rotational thromboelastometry may predict bleeding severity, measured as the volume of the postoperative chest tube output (CTO) and according to universal definition of perioperative bleeding (UDPB) scores [15].
(2) To evaluate the changes in platelet reactivity and viscoelastic blood properties monitored pre- and postoperatively using HPOCT and to propose an alternative therapeutic approach in a subgroup of patients with postoperative platelet hyperactivity coupled with a shift towards hypercoagulability, as assessed by rotational thromboelastometry (ROTEM).

## 2. Materials and Methods

**Figure 1:**
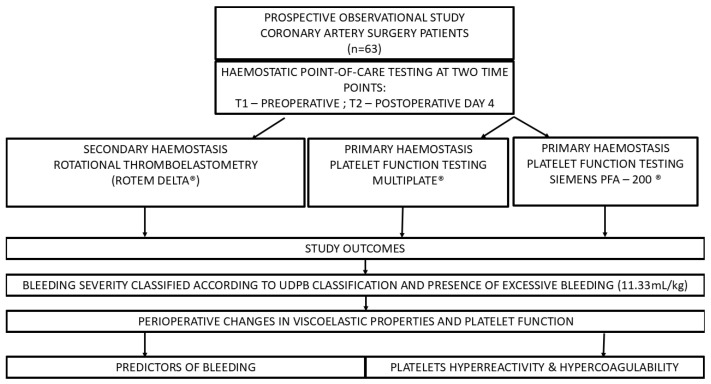
Study flow chart.

The study was conducted in a prospective observational fashion. Patients undergoing elective coronary artery bypass graft surgery in the period from February 2015 to June 2015 (CABG) were enrolled (Figure 1).

### 2.1. Blood Sampling

Blood samples for testing platelet function and conducting ROTEM measurements were collected at two different times: on the day of surgery using a central venous catheter (T1) and on the fourth postoperative day (POD 4) (T2). Blood for the Multiplate tests was drawn into 4 mL BD Vacutainer^®^ plastic tubes coated with heparin (Lithium Heparin 68 I.U.). For Siemens PFA-200 Innovance, blood was collected in 1.8 mL Vacutainer^®^ plastic tubes containing sodium citrate (0.109 Molar/3.2% citrate concentration). Similarly, blood for the ROTEM^®^ tests was collected in 1.8 mL sodium citrate (0.109 Molar/3.2% citrate concentration) Vacutainer^®^ plastic tubes. All measurements were conducted by the same individual, who was not directly involved in patient care. After blood withdrawal, Multiplate^®^ samples were allowed to rest for 30 min before testing, while ROTEM tests were conducted immediately following blood collection.

The hemostatic properties of the blood samples were evaluated using three distinct point-of-care (POC) devices. Two platelet function analyzers were employed:

(1) The aggregation of whole blood was measured using an impedance aggregometer (Multiplate^®^ analyzer, Roche, Basel, Switzerland). A comprehensive explanation of this method has already been published [16]. In summary, Multiplate^®^ operates on the principle that platelets remain non-aggregated in their resting state but reveal surface receptors upon activation, enabling them to clump together at sites of vascular injury and on artificial surfaces. When platelets adhere to the sensor wires of the Multiplate^®^ device, they increase the electrical impedance between the wires, which is continuously monitored. The rise in impedance is quantified in arbitrary area under the curve (AUC) units, which is recognized as the most diagnostically significant parameter [16]. Platelet aggregation was assessed following stimulation with arachidonic acid at a final concentration of 0.5 mM (using the ASPI test to evaluate the effect of Aspirin) and with adenosine diphosphate (ADP) at a final concentration of 6.4 µM (utilizing the ADP test to assess the impact of thienopyridines, such as clopidogrel) [16].
(2) The Siemens INNOVANCE^®^ PFA-200 System with the following assays: PFA Collagen/EPI test, PFA Collagen/ADP test, INNOVANCE^®^ PFA P2Y test.

The INNOVANCE^®^ PFA-200 System provides an automated assessment of platelet dysfunction. It measures the process of primary hemostasis and aids in the rapid detection of platelet dysfunction. The system measures platelet plug formation in a small whole-blood sample (800 μL) and reports a “closure time” in 5–8 min.

Principle of the method: The PFA Systems allow for a rapid evaluation of platelet function in small samples of citrated whole blood based on the work described by Kratzer et al. [17,18]. The single PFA test cartridges consist of a number of integrated parts including a capillary, a sample reservoir, and a biochemically active membrane with a central circular aperture. Citrated whole blood is aspirated from the sample reservoir through the capillary and the aperture, which expose platelets to high shear flow conditions. The membrane is collagen-coated, a subendothelial protein generally believed to be the initial matrix for platelet attachment. The attachment of platelets to collagen is thought to trigger the initial physiologic stimulus for platelet activation. In addition, the membrane is coated with either epinephrine or ADP, which are other physiologic agonists that, along with collagen, are widely used to activate platelets in functional testing. At the beginning of a PFA test, the trigger solution is dispensed to wet the membrane. During the test, platelets adhere to the collagen-coated membrane [17,18]. Then, similar to aggregometry [19], platelets become activated and release their granule contents upon contact with agonists such as ADP or epinephrine [19]. The release of granule contents is followed by adherence of platelets to each other to form aggregates. As a measure of platelet function in the PFA Systems, the process of platelet aggregation builds a platelet thrombus at the aperture, thereby gradually diminishing and finally arresting the blood flow [20]. In optical aggregometry, platelet function is assessed by aggregate formation detected by changes in light transmittance. The PFA Systems determine the time from the start of the test until the platelet plug occludes the aperture, and report that time interval as the Closure Time (CT). The CT is an indicator of platelet function in the analyzed whole-blood sample. Platelet plug formation in the PFA Systems is affected by low platelet counts and/or activity, a reduced plasma level of von Willebrand factor, and additionally by a decreased hematocrit because of the flow process [20].

The viscoelastic properties of blood were evaluated using the ROTEM^®^ delta (TEM Innovations GmbH, Munich, Germany) rotational thromboelastometry system. The ROTEM procedure was conducted in accordance with the manufacturer’s guidelines, utilizing the equipment and kits supplied by TEM Innovations GmbH. The following kits were utilized: the intrinsically activated coagulation (InTEM) and the extrinsically activated coagulation (ExTEM) kits, which promote clot formation via the intrinsic pathway and the extrinsic, tissue-factor-dependent pathway, respectively. The HepTEM assay (heparinase-modified ROTEM) triggers coagulation through the intrinsic pathway. Additionally, FibTEM is a test that assesses the role of fibrinogen, based on the ExTEM methodology, but includes cytochalasin D to prevent platelet contributions to clot firmness, thereby isolating the effect of fibrinogen on clot firmness. Digital data processing yields several key variables: clotting time (CT), which measures the duration from the start of measurement to the initiation of clotting; clot formation time (CFT), which is the time taken from the onset of clotting to when the clot firmness reaches an amplitude of 20 mm; and A 10, A 20, A 30, which represent clot firmness amplitudes recorded at 10, 20, and 30 min after the CT, respectively.

POC tests were performed simultaneously at two different time points: (T1) before surgery and on (T2) postoperative day 4, respectively.

The primary outcome of the study was defined as the amount of perioperative bleeding and transfusion requirements, classified according to the UDPB score [15]. Additionally, we classified patients with respect to the presence of excessive bleeding according to the 24 h chest tube output (CTO). Chest tubes remain in the thoracic cavity until drainage tapers to less than 100 mL over the previous 8 h. The total time with chest tubes varies between patients but all patients have chest tubes in place over a period of the first 24 h, and usually after 24 h, the chest tubes are taken out. Our rationale was to measure CTO over the period of the first 24 postoperative hours. A 24 h CTO is expressed in mL/kg of body weight. Although there are some definitions of abnormal blood loss in cardiac surgery, we have made our own definition of excessive postoperative bleeding to adjust postoperative CTO to our study group. Such a definition represents a more reliable correlation, and it is not influenced by surgical, perfusionist, and anesthetic techniques. Patients were characterized as bleeders if their CTO in the first 24 h (mL/kg) exceeded the 75th percentile of distribution [21]. A 24 h CTO exceeding 11.33 mL/kg of body weight was considered to be excessive bleeding [21]. Our working group provided this definition in previous research. A comparable definition has already been documented in the literature [22]. Using this cutoff value, we divided patients with respect to secondary outcome measures into groups with and without excessive bleeding in the early postoperative period.

The UDPB score is based on 9 events occurring during surgery or within the first postoperative day: delayed sternal closure, postoperative CTO, PRBC transfusion, FFP transfusion, PLT transfusion, cryoprecipitate transfusion, use of factor concentrates, use of recombinant activated factor VII (rFVIIa), and surgical re-exploration [15]. There are 5 perioperative UDPB classes, divided in accordance with the severity of bleeding, regardless of surgical or hemostasis issues. The presence of all attributes within a class is not necessary, the presence of the single worst attribute is sufficient to assign a patient in a particular bleeding class; hence, the worst-parameter principle is applied throughout the bleeding classification system [15].

### 2.2. Perioperative Management

All patients received the same anesthetic and perfusion teams and were admitted at least one day prior to surgery. The procedure was carried out using a standardized technique, with a median sternotomy approach employed in all cases. All measurements were conducted by a research fellow who was not involved in patient treatment. The nursing staff, anesthesiologists, and surgeons responsible for patient care were unaware of the HPOCT results. The surgeries took place in a single unit utilizing standard surgical methods. Surgical bleeding was managed using diathermy and bone wax. The cardiopulmonary circuit included the Medtronic Affinity Trillium membrane oxygenator, a venous reservoir, and PVC tubing (Medtronic, Minneapolis, MN, USA), along with a Stoeckert III roller pump (Stoeckert, Munich, Germany). Targeted flow rates were set at 2.2 L/min/m^2^ with an average blood pressure of 60 mmHg. Cardiac arrest was achieved via cold blood cardioplegia. Systemic heparinization was administered, aiming for an activated clotting time (ACT) of 480 s, followed by complete reversal with protamine after decannulation. A 1 g dose of tranexamic acid was given at the onset of anesthesia and following protamine administration. Inotropic support was initiated to maintain a cardiac index above 2.2 L/min/m^2^. Weaning from the cardiopulmonary bypass (CPB) began once the patient’s heart rhythm stabilized and normothermia was restored. Blood was returned to the CPB circuit via cardiotomy suction. Packed red blood cells (PRBCs) were transfused if the hematocrit fell below 20% during CPB or below 25% after CPB cessation, or in cases of significant bleeding. Volume replacement in the intensive care unit was provided as needed by the attending physician using hydroxyethyl starch 6% 130/0.4 and lactated Ringer’s solution. PRBC transfusions were given based on the consultant anesthesiologist’s judgment. Fresh frozen plasma (FFP) was administered in situations where prothrombin time was prolonged (below 45% of normal activity) or based on clinical discretion from the consultant anesthesiologist.

### 2.3. Statistical Analysis

The Kolmogorov–Smirnov test was utilized to assess the normality of the distribution for all continuous variables. Spearman’s correlation coefficient was employed to evaluate the relationship between CTO during the first 24 h post-surgery and HPOCT parameters. The Mann–Whitney U test was used to determine if there were significant differences between the medians of a test variable across the two groups. The chi-square statistical test was conducted to compare the frequency distribution of observed categorical variables between the two groups. Receiver operating characteristic (ROC) analysis was performed to evaluate the predictive capacity of HPOCT parameters for the occurrence of excessive postoperative CTO [23]. A value of *p* < 0.05 was considered statistically significant. For statistical analysis we used MedCalc^®^ For Windows (Med-Calc Software Version 23.0.2, Broekstraat 52, 9030 Mariakerke, Belgium).

## 3. Results

The study recruited a total number of 63 patients undergoing elective isolated CABG in the period from 21 August 2014 to 18 December 2014. Basic demographic, procedural, outcome data, and HPOCT data are shown in Table 1, Table 2 and Table 3, respectively.

A total number of 2 out of 63 patients (3.2%) underwent re-exploration for excessive bleeding. None of the patients had delayed sternal closure (open chest) for the excessive bleeding in the study cohort.

When comparing patients with respect to the presence of excessive bleeding, we found the following results. Patients with excessive bleeding were found to have a significantly lower BMI (27.66 ± SD4.67 vs. 30.44 ±SD 3.68, *p* = 0.017). Even though the Multiplate^®^ ASPI and ADP tests were lower in the excessive bleeding group, the difference did not reach statistical significance (*p* = ns). Non-significant differences were noted for the procedural parameters (cardiopulmonary bypass and cross clamp time), as well (*p* = ns). Given the PRBC transfusion requirements, excessive bleeding patients were not just more frequently transfused (87.5% vs. 48.9%, *p* = 0.007) but were also transfused with a higher amount of PRBCs (1338.75 mL ± SD 1416.49 vs. 289.36 mL ± 373.07, *p* < 0.001).

We compared preoperative ROTEM parameters between patients with and without excessive bleeding. Patients with excessive postoperative bleeding had significantly lower clot firmness results preoperatively, as assessed by the FIBTEM test (FIBTEM MCF, 20.44 ± 4.27 mm vs. 24.22 ± 7.19 mm, *p* = 0.030; FIBTEM A10, 19.00 ± 3.98 mm vs. 22.35 ± 6.78 mm, *p* = 0.045; FIBTEM, A30 20.56 ± 4.24 mm vs. 24.33 ± 7.20 mm, *p* = 0.029). FIBTEM A30 significantly correlated with excessive bleeding (Correlation Coefficient Rho = −0.280, *p* = 0.028) (Table 4).

FIBTEM A10 also significantly correlated with excessive bleeding (Correlation Coefficient Rho = −0.256, *p* = 0.044). The same holds for FIBTEM MCF (Correlation Coefficient Rho = −0.279, *p* = 0.028) (Table 4).

**Figure 2:**
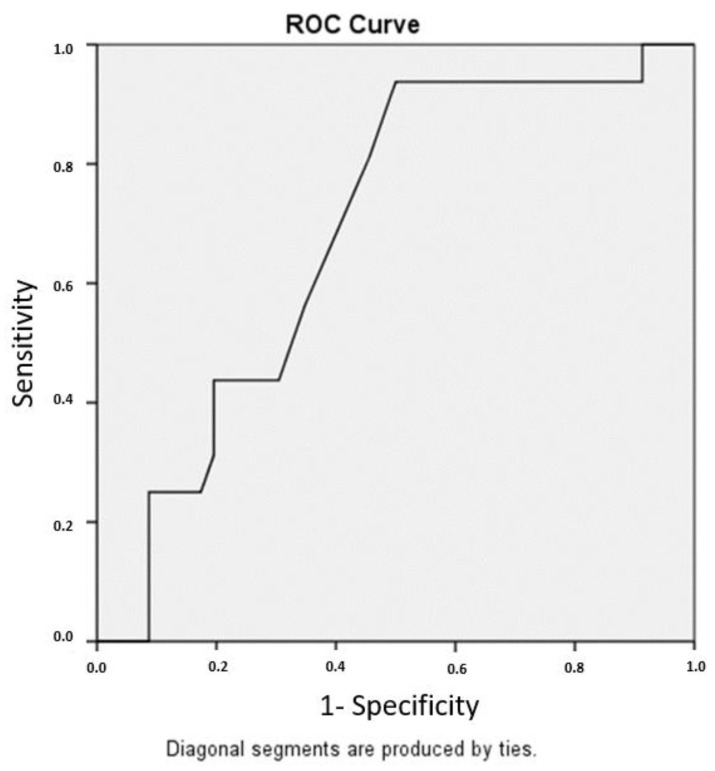
Receiver operating characteristics curve (ROC) analysis demonstrated that a preoperative FIBTEM A30 cut-off value below 23.49 mm predicted excessive bleeding (ROC AUC 68.4%, sensitivity 93.8%, and specificity 50%).

Regression analysis revealed FIBTEM A 30 as the strongest predictor of 24 h CTO. The calculated cut-off value (optimal Yi) was 23.49. (AUC 0.684, SE = 0.072, *p* = 0.029, 95% CI 0.544–0.824), as shown in Figure 2. To adjust this calculation to values obtained by ROTEM Delta in clinical practice (rounded numbers without decimals), we decided to define the cut-off value as 24 mm with a calculated sensitivity 93.8% and specificity 50%. The positive predictive value was 39.5%, whereas the negative predictive value was 95.8%.

For the Siemens PFA-200 parameters we found no difference in patients with and without excessive bleeding.

**Figure 3:**
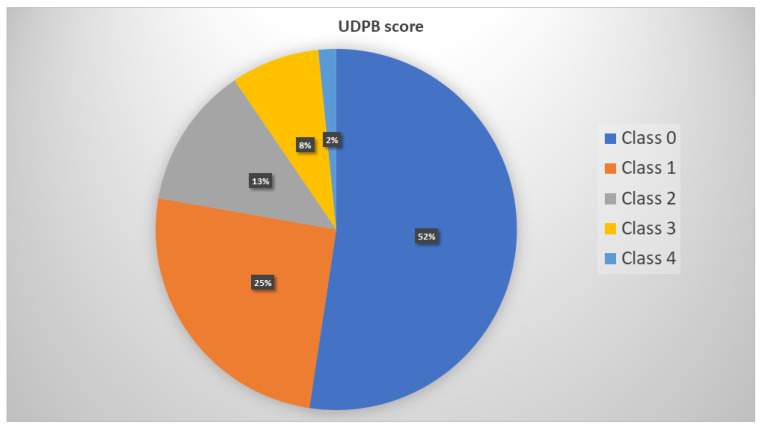
The distribution of the UDPB score bleeding severity levels among the study cohort. UDPB (universal definition of perioperative bleeding) classes: Class 0—insignificant bleeding; Class 1—mild; Class 2—moderate; Class 3—severe; Class 4—massive.

The UDPB score was used to classify patients according to the postoperative bleeding severity. The prevalence of different UDPB scoring levels is shown in Figure 3.

In our cohort, a proportion of 52% of patients had insignificant bleeding. Bearing in mind our study cohort size, as well as the distribution of UDPB classes, we divided the UDPB classes into two groups, group 1 with Class 0 (insignificant bleeding) and Group 2 encompassing Classes 1, 2, 3, and 4 (representing all but insignificant bleeding), respectively. We found good agreement between bleeding severity according to our definition of excessive bleeding and the UDPB score (*p* < 0.001). In patients without excessive bleeding according to our definition, a proportion of 66% had insignificant bleeding (UDPB Class 0).

Regarding preoperative hemoglobin (Hb) levels, patients in Group 2 had significantly lower Hb values (131.83 ± 15.69 g/L vs. 141.15 ± 13.63 g/L, *p* = 0.042).

Regarding the platelet function testing results, patients in Group 2 showed significantly lower preoperative Multiplate ASPI test results (19.4 ± 10.7 AU vs. 35.2 ± 22.2 AU, *p* = 0.01) and a higher proportion of Aspirin-responders (76.7% vs. 42.4%, *p* = 0.006).

**Figure 4:**
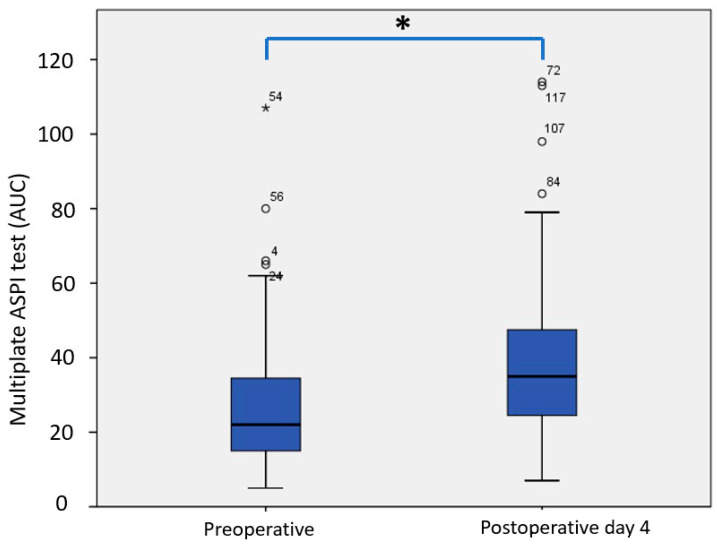
Comparison between preoperative Multiplate ASPItest values (27.7 ± 19.3 AU) and postoperative values at POD4 (39.9 ± 23.4 AU) (*p* = 0.001). * Statistically significant.

Furthermore, changes between preoperative values and values on postoperative day 4 are also important. We analyzed perioperative changes in all three HPOCT tests used. When analyzing paired sample correlations (correlations between preoperative values and postoperative values on POD 4) in the Multiplate assays, we found significant correlations in both ASPItest (r = 0.268, *p* = 0.033) and ADPtest (0.272, *p* = 0.031). Paired samples matching revealed significantly higher ASPItest values on POD4 compared to the preoperative values (39.9 ± 23.4 AU vs. 27.7 ± 19.3 AU, *p* = 0.001) (Figure 4).

The same trend was noticed for the ADPtest, as well. However, the difference did not reach statistical significance (79.6 ± 33 AU vs. 74.4 ± 22.7 AU, *p* = 0.236).

Considering the Siemens PFA-200 assays, we found non-significant correlations between pre- and postoperative values. Only the P2Y assay was significantly higher at POD 4 compared to the preoperative results (82.3 ± 39.3 vs. 64.3 ± 17.9, *p* = 0.01).

Both Multiplate and Siemens PFA-200 assess primary hemostasis. The changes in primary hemostasis should be put into the context of the changes in secondary hemostasis. Strong and significant differences have been observed in the ROTEM parameters. The ROTEM parameters at POD 4 were consistently and significantly different compared to the preoperative results suggesting a clear shift towards hypercoagulability.

## 4. Discussion

In our study, patients with excessive bleeding had significantly lower preoperative clot firmness results, as assessed by the FIBTEM test. These results are in line with our previous findings in patients undergoing all kinds of cardiac surgery procedures [24]. This suggests that we can not only predict the bleeding risk in the general cardiac surgery population [24], but also specifically in patients who undergo isolated GABG which is considered to be a less complex procedure with a lower risk for excessive bleeding. FIBTEM A30 significantly correlated with excessive bleeding (Correlation Coefficient Rho = −0.280, *p* = 0.028) (Table 4). FIBTEM A10 also significantly correlated with excessive bleeding (Correlation Coefficient Rho = −0.256, *p* = 0.044). The same holds for FIBTEM MCF (Correlation Coefficient Rho = −0.279, *p* = 0.028) (Table 4). However, the correlation between FIBTEM A20 and excessive bleeding did not reach statistical significance (correlation coefficient −0.238, *p* = 0.062). Given the Rho value of −0.238, we may assume that the sample size effect herein could have made this correlation significant if more patients had been recruited to the study. In contrast to the ROTEM FIBTEM assay, the EXTEM assay parameters consistently reported a lack of correlation with excessive bleeding (Table 4). Given the relationship between the EXTEM and FIBTEM assays, we may assume that platelet contribution to clot firmness certainly does not increase the association between clot firmness and excessive bleeding. Recently, our working group developed a SHOULD-NOT-BLEED score [25], a Windows-based application based on a statistical model developed to stratify patients according to their bleeding risk [25]. In the SHOULD-NOT-BLEED score, the Fibrinogen level, as assessed by the conventional laboratory Clauss method, was identified as a predictor of excessive bleeding [25]. Now, with the HPOCT ROTEM FIBTEM assay, the model may be improved regarding its predictive value. Our data suggest that the addition of the ROTEM FIBTEM test to the existing model could improve its predictive performance. The negative predictive value of 95.8% may definitively contribute to understanding who should not bleed after surgery. Preoperative FIBTEM test results may have a great clinical value as a predictor for bleeding and may shift our focus towards plausible causes for bleeding if excessive bleeding occurs following CABG surgery. However, this must be validated by prospective multicenter observational trials. Nevertheless, this raises the question whether preoperative supplementation with a fibrinogen concentrate can reduce postoperative blood loss. Jeppsson et al. [26] evaluated preoperative supplementation with a fibrinogen concentrate in cardiac surgery in a randomized controlled trial. Here, preoperative supplementation with 2 g fibrinogen concentrate did not significantly reduce postoperative bleeding in CABG patients without hypofibrinogenemia [26]. However, these results may be different if we focus on CABG patients with (1) a calculated higher risk of bleeding, as assessed by the SHOULD-NOT-BLEED score coupled with (2) low preoperative FIBTEM test results (A30 < 24 mm). A randomized controlled trial recruiting this specific patient population may show positive results based on the hypothesis that preoperative administration of Fibrinogen concentrate may be more effective in patients with preoperative FIBTEM A30 < 24 mm and a calculated high risk for bleeding.

When analyzing the Multiplate ASPItest results in the context of different UDPB classes, patients in UDPB Class 0 (insignificant bleeding) had significantly higher ASPItest results (35.24 ± 22.24 AU vs. 19.43 ± 10.74 AU, *p* = 0.001) compared to patients in Group 2 (clustered classes 1, 2, 3, and 4, respectively).

Considering perioperative changes in the HPOCT results, we found significant changes in all three HPOCT results. However, not all assays showed significantly different results when the respective parameter pairs were matched.

Regarding the Multiplate tests, ASPItest was significantly higher at POD 4 (Figure 3) relative to the preoperative values (39.87 ± 23.42 AU vs. 27.71 ± 19.30 AU, *p* = 0.001). These results suggest platelet hyperreactivity in the early postoperative period. In patients with such an increase in platelet activity, an adequate platelet inhibitory response to Aspirin at POD 4 was only found in 39.7% of patients. In contrast, we found that preoperatively, 58.7% of patients had an adequate platelet inhibitory response to Aspirin. This difference is even more important considering that patients were preoperatively exposed to 100 mg Aspirin once daily, whereas the postoperative dose of Aspirin was 300 mg once daily.

In this study, we conducted a thorough analysis of perioperative changes in both primary and secondary hemostasis. Our findings indicate that the increase in platelet reactivity during the perioperative period is associated with ROTEM parameters, which point to a trend toward hypercoagulability. Furthermore, our research team has created a concept for a personalized strategy regarding the management of perioperative antiplatelet therapy administration and discontinuation, utilizing multiple electrode aggregometry for patients undergoing coronary artery bypass grafting (CABG) [27]. This marked the initial effort to establish a “therapeutic window” based on platelet function testing, which would guide the management of administering or discontinuing APT during the perioperative period [27].

The idea of a “one-size-fits-all” approach to managing perioperative APT administration appears to be ineffective. Developing a personalized APT administration and discontinuation management system based on HPOCT may prove to be more advantageous. The use of ROTEM may allow for identifying patients with hypercoagulability in the early postoperative period with an increased risk of ischemic events such as myocardial infarction or stroke. Improved anticoagulation in patients with detected postoperative hypercoagulability may be beneficial. However, such an approach needs to be validated in prospective interventional studies or RCTs. This idea signifies a potential advancement in personalized patient care and serves as a foundation for additional interventional trials aimed at evaluating and confirming the effects of its implementation on clinical patient outcomes. A consideration of ROTEM test results is not only important in the intra- and postoperative period but also in the preoperative period to assess the bleeding risk more reliably, e.g., by using a FIBTEM test cutoff value and maybe even considering prophylactic administration of the fibrinogen concentrate in high-risk cases. However, this must be assessed in future RCTs. Meesters et al. evaluated the predictability of bleeding in cardiac surgery patients using ROTEM tests [28]. This study showed that the positive predictive value of preoperative and postoperative thromboelastometric results was poor (0–22%). However, the negative preddictive value was high (89–94%) [28]. The authors concluded that ROTEM does not predict which patients are at risk of major postoperative bleeding [28]. However, our research shows that ROTEM results may be used to stratify patients according to their bleeding risk. A thorough understanding of the research methodology is needed to better implement the available results [21]. We agree that the positive predictive value is low; however, the negative predictive value is consistently high throughout studies evaluating HPOCT in bleeding risk prediction [21]. Even though we do not obtain a clear answer to the question “who will bleed” due to the low PPV, we receive very valuable information regarding the question “who should not bleed”, or at least should not bleed due to the hemostatic alterations [21]. This information is very important and useful and may guide our hemostatic management towards bleeding risk stratification, identification of patients that should not bleed, and in the case of persisting bleeding, may shift our focus towards issues that may lead to the progression of bleeding [21]. Here, the low FIBTEM test results may result in prophylactic Fibrinogen concentrate administration.

## 5. Conclusions

Hemostatic POCT devices assessing both primary (platelets function) and secondary (coagulation) hemostasis are useful tools to stratify patients according to the bleeding risk, even in low-risk populations such as patients undergoing CABG surgery.

Identification of lower than cut-off preoperative FIBTEM test results may allow for bleeding risk stratification and for preoperative prevention measures to decrease the risk for bleeding, such as fibrinogen concentrate administration, as a preemptive measure or as a first line therapy in case of excessive bleeding.

Significant perioperative changes are detected using HPOCT devices, showing a trend towards higher platelet reactivity in the postoperative phase coupled with changes in secondary hemostasis suggesting hypercoagulability. These results suggest further development of the concept of a personalized approach towards APT administration, encompassing considerations for the administration of anticoagulant medications even in the early postoperative period. However, such an approach needs to be validated in prospective interventional trials and RCTs. Pooling of the evidence in this research field is hampered by numerous methodological considerations [21]. Therefore, multicentric trials would be adequate to overcome the differences between different research groups [21]. A standardized approach to defining outcomes (such as postoperative bleeding amount) is mandatory. Accordingly, we suggest using the UDPB score [15] in such studies to standardize the outcomes and decrease contradictions in results [28] for the prediction of bleeding using POCT [21]. Our results may serve as an impetus for further research and update of the current concepts for a personalized approach in preoperative bleeding risk assessment, targeted transfusion in bleeding patients, and a personalized approach towards perioperative APT administration/discontinuation and management.

## Figures and Tables

**Table 1:** Basic demographic data (continuous variables). Abbreviations: BMI—body mass index; BSA—body surface area; CTO—chest tube output; INR—international normalized ratio; LVEF—left ventricular ejection fraction; RBCC—red blood cells concentrate.

	N	Minimum	Maximum	Mean	Std. Deviation
EuroSCORE2	63	0.55	8.13	2.25	1.69
Weight (kg)	63	46	131	89.01	16.1
Height (cm)	63	150	197	172.7	8.77
BMI (kg/m^2^)	63	20.4	40.4	29.7	4.10
BSA (m^2^)	63	1.38	2.63	2.06	0.23
Age (years)	63	47	80	65	8.15
LVEF (%)	63	30	78	54.9	10.3
Preoperative Red Blood Cells Count (million/mm^3^)	63	2.88	5.54	4.53	0.50
Preoperative Hemoglobin (g/L)	63	84	167	136.7	15.3
Preoperative Hematocrit (%)	63	0.23	0.47	0.40	0.05
Preoperative Platelets Count (10^9^/L)	62	104	428	211.9	64
Preoperative INR	63	0.85	4.37	1.03	0.43
Preoperative Fibrinogen (g/L)	59	2.40	8.60	4.16	1.12
Aortic Cros Clamp Time (min)	49	0	98	53.2	16.6
Cardiopulmonary Bypass Time (min)	49	43	116	76.1	19.2
CTO 24 h (mL)	63	200	7650	930.2	976.6
CTO24 h (mL/kg)	63	1.74	67.1	10.6	9.18
CTO total amount (mL)	63	85	9920	1501.4	1720
CTO total amount (mL/kg)	63	0.79	108.9	17.1	18.1
Total RBCC transfusion (mL)	63	0	5720	555.9	894.8

**Table 2:** Basic demographic data (Categorical Parameters). Abbreviations: ASA—acetylsalicylic acid; AMI—acute myocardial infarction; DM2—diabetes mellitus type 2; IDDM2—insulin dependent diabetes mellitus type 2; LMCA—left main coronary artery; LMWH—low molecular weight heparin; POD 4—postoperative day 4; UDPB—universal definition of perioperative bleeding; RBC—packed red blood cells.

			Number	Percent	Valid Percent	Cumulative Percent
Gender	Valid	M	56	88.9	88.9	88.9
		F	7	11.1	11.1	100.0
		Total	63	100.0	100.0	
DM2	Valid	0	43	68.3	68.3	68.3
		1	20	31.7	31.7	100.0
		Total	63	100.0	100.0	
IDDM2	Valid	0	16	25.4	80.0	80.0
		1	4	6.3	20.0	100.0
		Total	20	31.7	100.0	
	Missing	System	43	68.3		
	Total		63	100.0		
Smoking	Valid	0	19	30.2	30.2	30.2
		1	44	69.8	69.8	100.0
		Total	63	100.0	100.0	
Preoperative AMI	Valid	0	48	76.2	76.2	76.2
		1	15	23.8	23.8	100.0
		Total	63	100.0	100.0	
LMCA Disease	Valid	0	38	60.3	60.3	60.3
		1	25	39.7	39.7	100.0
		Total	63	100.0	100.0	
Triple vessel disease	Valid	0	19	30.2	30.2	30.2
		1	44	69.8	69.8	100.0
		Total	63	100.0	100.0	
Postoperative inotropes	Valid	0	47	74.6	74.6	74.6
		1	16	25.4	25.4	100.0
		Total	63	100.0	100.0	
RBC transfusion	Valid	0	26	41.3	41.3	41.3
		1	37	58.7	58.7	100.0
		Total	63	100.0	100.0	
Postoperative FA	Valid	0	45	71.4	71.4	71.4
		1	18	28.6	28.6	100.0
		Total	63	100.0	100.0	
POD 4 ASA responder	Valid	0	38	60.3	60.3	60.3
		1	25	39.7	39.7	100.0
		Total	63	100.0	100.0	
Postoperative Reexploration	Valid	0	61	96.8	96.8	96.8
		1	2	3.2	3.2	100.0
		Total	63	100.0	100.0	
UDPB score	Valid	0	33	52.4	52.4	52.4
		1	16	25.4	25.4	77.8
		2	8	12.7	12.7	90.5
		3	5	7.9	7.9	98.4
		4	1	1.6	1.6	100.0
		Total	63	100.0	100.0	
ASA responder	Valid	0	26	41.3	41.3	41.3
		1	37	58.7	58.7	100.0
		Total	63	100.0	100.0	
POD 4 ASA responder	Valid	0	38	60.3	60.3	60.3
		1	25	39.7	39.7	100.0
		Total	63	100.0	100.0	
Preoperative LMWH	Valid	0	20	31.7	31.7	31.7
		1	43	68.3	68.3	100.0
		Total	63	100.0	100.0	
Preoperative clopidogrel	Valid	0	51	81.0	81.0	81.0
		1	12	19.0	19.0	100.0
		Total	63	100.0	100.0	
Preoperative ASA	Valid	0	3	4.8	4.8	4.8
		1	60	95.2	95.2	100.0
		Total	63	100.0	100.0	

**Table 3:** Platelet function testing and rotational thromboelastometry data for the study cohort at both preoperative and postoperative (postoperative day 4—POD4) timepoints.

	N	Minimum	Maximum	Mean	Std. Deviation
	**Multiplate ^®^ Whole Blood Impedance Aggregometry**				
Preoperative Multiplate ASPI test AUC [AU]	63	5	107	27.7	19.3
Preoperative Multiplate ADP test AUC [AU]	63	26	126	74.4	22.7
POD4 Multiplate ASPI test AUC [AU]	63	7	114	39.9	23.4
POD4 Multiplate ADP test AUC [AU]	63	19	146	79.6	33
	**Siemens PFA-200**				
Preoperative COL_EPI_ratio	37	85	293	156.5	53.2
Preoperative COL_ADP_ratio	58	52	296	89.7	31.8
Preoperative P2Y	59	43	224	69.2	28.2
POD4 COL_EPI_ratio	46	83	242	136.7	36.3
POD4 COL_ADP_ratio	45	61	620	108	82.8
POD4 P2Y	50	47	218	82.3	38.1
	**ROTEM Delta^®^ Rotational Thromboelastometry**				
Preoperative EXTEM CT	62	37	243	73.2	39.9
Preoperative EXTEM CFT	62	31	249	70.9	27.5
Preoperative EXTEM alfa	62	60	84	76.4	3.5
Preoperative EXTEM MCF	62	45	84	68.8	5.3
Preoperative EXTEM A10	62	33	82	62.7	6.5
Preoperative EXTEM A20	62	41	84	68.0	5.7
Preoperative EXTEM A30	62	44	84	68.5	5.4
Preoperative INTEM CT	62	12	329	179.8	40.1
Preoperative INTEM CFT	62	29	317	77.6	40.5
Preoperative INTEM alfa	62	55	84	75.2	4.4
Preoperative INTEM MCF	62	42	80	64.4	5.2
Preoperative INTEM A10	62	29	77	58.3	6.3
Preoperative INTEM A20	62	38	80	63.9	5.5
Preoperative INTEM A30	62	41	79	63.7	5.3
Preoperative FIBTEM CT	62	34	133	63.1	23.7
Preoperative FIBTEM alfa	59	51	83	73.8	6.3
Preoperative FIBTEM MCF	62	13	49	23.2	6.7
Preoperative FIBTEM A10	62	12	46	21.5	6.3
Preoperative FIBTEM A20	62	13	48	22.8	6.7
Preoperative FIBTEM A30	62	13	49	23.4	6.7
POD4 EXTEM CT	61	41	164	74.9	28.6
POD4 ECTEM CFT	61	33	233	62.2	27.6
POD4 EXTEM alfa	61	51	83	77.9	4.6
POD4 EXTEM MCF	61	48	81	71.7	5.4
POD4 EXTEM A10	61	35	78	66.4	6.7
POD4 EXTEM A20	61	44	81	71.3	5.7
POD4 EXTEM A30	61	47	80	71.2	5.5
POD4 INTEM CT	62	97	356	159.3	37
POD4 INTEM CFT	62	39	306	66.3	34.9
POD4 INTEM alfa	62	49	82	77.2	4.5
POD4 INTEM MCF	62	42	78	67	5.8
POD4 INTEM A10	62	30	75	62	7
POD4 INTEM A20	62	38	78	66.8	6.2
POD4 INTEM A30	62	41	78	66.1	6.1
POD4 FIBTEM CT	62	30	132	61.1	17.4
POD4 FIBTEM alfa	61	45	82	76.4	5.3
POD4 FIBTEM MCF	62	12	81	33	9.5
POD4 FIBTEM A10	62	11	51	30.2	7.1
POD4 FIBTEM A20	62	12	53	31.9	7.2
POD4 FIBTEM A30	62	13	53	32.3	7.2

**Table 4:** Spearman’s correlation test between preoperative point-of-care Siemens PFA-200, Multiplate, and ROTEM Delta assays and the presence of excessive bleeding.

	(Spearmans Rho)	Excessive Bleeding
Multiplate ASPI test	Correlation Coefficient	−0.166
	Sig. (2-tailed)	0.195
Multiplate ADP test	Correlation Coefficient	−0.050
	Sig. (2-tailed)	0.696
COL_EPI ratio	Correlation Coefficient	−0.108
	Sig. (2-tailed)	0.523
COL_ADP ratio	Correlation Coefficient	0.064
	Sig. (2-tailed)	0.636
P2Y	Correlation Coefficient	−0.014
	Sig. (2-tailed)	0.916
EXTEM CT	Correlation Coefficient	0.184
	Sig. (2-tailed)	0.151
EXTEM CFT	Correlation Coefficient	−0.205
	Sig. (2-tailed)	0.110
EXTEM alfa	Correlation Coefficient	0.152
	Sig. (2-tailed)	0.237
EXTEM MCF	Correlation Coefficient	−0.021
	Sig. (2-tailed)	0.873
EXTEM A10	Correlation Coefficient	0.056
	Sig. (2-tailed)	0.666
EXTEM A20	Correlation Coefficient	0.014
	Sig. (2-tailed)	0.911
EXTEM A30	Correlation Coefficient	−0.037
	Sig. (2-tailed)	0.773
INTEM CT	Correlation Coefficient	−0.144
	Sig. (2-tailed)	0.263
INTEM CFT	Correlation Coefficient	−0.099
	Sig. (2-tailed)	0.444
INTEM alfa	Correlation Coefficient	0.107
	Sig. (2-tailed)	0.408
INTEM MCF	Correlation Coefficient	−0.126
	Sig. (2-tailed)	0.327
INTEM A10	Correlation Coefficient	−0.061
	Sig. (2-tailed)	0.638
INTEM A20	Correlation Coefficient	−0.144
	Sig. (2-tailed)	0.265
INTEM A30	Correlation Coefficient	−0.145
	Sig. (2-tailed)	0.260
FIBTEM CT	Correlation Coefficient	0.201
	Sig. (2-tailed)	0.117
FIBTEM alfa	Correlation Coefficient	−0.034
	Sig. (2-tailed)	0.798
FIBTEM MCF	Correlation Coefficient	−0.279
	Sig. (2-tailed)	0.028
FIBTEM A10	Correlation Coefficient	−0.256
	Sig. (2-tailed)	0.044
FIBTEM A20	Correlation Coefficient	−0.238
	Sig. (2-tailed)	0.062
FIBTEM A30	Correlation Coefficient	−0.280
	Sig. (2-tailed)	0.028

## Data Availability

The original contributions presented in this study are included in the article. Further inquiries can be directed to the corresponding author(s).

## Abbreviations

APT—antiplatelet therapy; ADP—adenosine di-phosphate test assay; ASPI—arachidonic acid test assay; AU—aggregation units; AUC—area under curve; BMI—body mass index; BSA—body surface area; CABG surgery—coronary artery bypass graft surgery; CFT—clot formation time; CT—clotting time or closure time; CTO—chest tube output; CPB—cardiopulmonary bypass; DM2—diabetes mellitus type 2; FFP—fresh frozen plasma; HPOCT—hemostatic point-of-care testing; IDDM2—insulin dependent diabetes mellitus type 2; LVEF—left ventricular ejection fraction; POCT—point-of-care testing; PRBC—packed red blood cells; ROC curve—receiver operating characteristics curve; ROTEM—rotational thromboelastometry; RPR—Residual platelet reactivity; UDPB—universal definition of perioperative bleeding.
